# Ileum Obstruction Following Appendix Adhesion Band after Sleeve Surgery: A Case Report and Review of Literature

**DOI:** 10.31661/gmj.v13i.3353

**Published:** 2024-05-15

**Authors:** Fezzeh Elyasinia, Hamid Zaferani Arani, Amir Reza Radmard, Ahmadreza Soroush, Fayez Farzamnia, Aydin Yaghobi Notash, Reza Eslamian, Khosrow Najjari, Hossein Zabihi Mahmoudabadi, Mohammad Hadi Niakan

**Affiliations:** ^1^ Department of Surgery, Tehran University of Medical Sciences, Tehran, Iran; ^2^ Department of Radiology, School of Medicine, Tehran University of Medical Sciences, Tehran, Iran; ^3^ Trauma Research Center, Shahid Rajaee (Emtiaz) Trauma Hospital, Shiraz University of Medical Sciences, Fars Province, Shiraz, Iran

**Keywords:** Sleeve Surgery, Intestinal Obstruction, Ileum, Appendicitis

## Abstract

Sleeve gastrectomy is a popular surgical procedure for weight loss. Although it is basically a safe surgery; however, it can lead to serious complications such as intestinal obstruction. The present report describes a 55-year-old woman who attended with complications of abdominal pain and vomiting that underwent laparoscopic exploratory intervention and ileum obstruction due to adherent bands of the appendix was considered as final diagnosis.

## Introduction

Surgical procedures on the gastrointestinal tract have become increasingly popular in the last decade, with sleeve gastrectomy being one of the most commonly performed bariatric surgeries [1]. Although the rate of gastric surgery has increased in both developing and developed countries, its prevalence can vary depending on factors such as healthcare infrastructure, cultural attitudes towards weight loss, access to medical facilities, and government policies [2][3].

Evidence shows that bariatric surgeries are generally safe and effective; however, it is not without potential complications [4]. Ileum obstruction subsequent to abdominal surgeries, particularly bariatric procedures such as sleeve gastrectomy is relatively rare [5]. To the best of our knowledge, ileum obstruction following sleeve surgery due to the adhesive bundles of the appendix was not previously reported. In this report, we introduced a patient who presented with symptoms of intestinal obstruction after gastric surgery.

## Case Presentation

**Figure-1:**
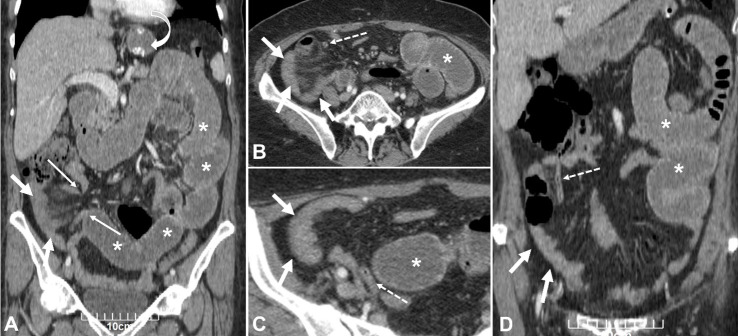
Figure 1. CT scan of the patient in coronal (A and D) and axial (B and C) views. Collapsed herniated loops of distal ileum are seen at RLQ (arrows) with mild adjacent mesenteric fluid and fat haziness associated with upstream small bowel dilatation (asterisks). An elongated loop of the appendix (dotted arrows) is noted arising from a highly located cecum extending down to the sac of hernia on the right side of the pelvis without appendiceal wall thickening. Two focal areas of luminal narrowing with beak-like appearance (thin arrows) are depicted in the distal ileum at the neck of the internal hernia with dilated upstream and collapsed downstream ileum showing a closed loop appearance. Signs of previous gastric sleeve surgery are seen in the upper abdomen (curved arrow).

**Figure-2:**
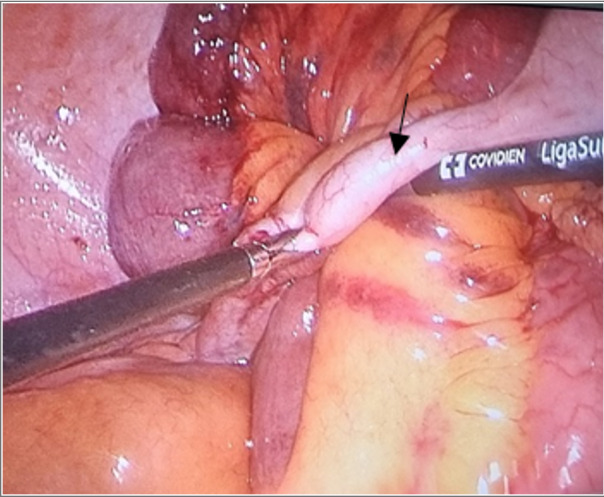
Figure 2. Performing surgery through laparoscopy. An elongated loop of the appendix (arrows) is noted arising from the highly located cecum extending down to the sac of hernia on the right side of the pelvis without appendiceal wall thickening

A 55-year-old woman was attended to our emergency ward (Shariati Hospital, Tehran, Iran) the chief complication of sudden generalized abdominal pain in the epigastric region and vomiting for the last two days. Also, she reported constipation and loss of appetite. Her past medical history revealed diabetes mellitus and hypertension that were controlled with oral agents. Past medical surgeries consist of cesarean section (15 years ago), cholecystectomy (15 years ago), sleeve surgery (14 months ago), and hemorrhoidectomy (15 years ago). On the physical exam, the abdomen was soft, and only diffuse tenderness particularly periumbilical was observed. The vital signs were within normal limits. There were no other abnormal findings in physical examinations. Considering our more probable differential diagnosis (i.e., intestinal obstruction/perforation/ appendicitis, and peptic ulcer), she underwent imaging studies.

Abdominal sonography showed no any abnormal results and there was no evidence of appendicitis. Hence, the computed tomography (CT) scan of the abdomen and pelvis was performed and revealed focal areas of luminal narrowing at the distal ileum as well as elongated the loop of the appendix and collapsed herniated loops of distal ileum (Figure-1).

Regarding the clinical presentation and history, a suspicion of small bowel obstruction was raised, and the patient underwent exploratory abdominal laparoscopy. Briefly, via general anesthesia, the patient was placed in the supine position and then Trendelenberg. once, ports have been placed. By using two-port No.10 (one 1cm above the umbilical and the other on epigastric) abdominal cavity was examined laparoscoply. Assisting ports can be a 5 mm right upper paramedian port for the surgeon’s left hand and a 5mm left anterior axillary line port for the assistant’s right hand. There were no any free fluids or perforation of the intestinal. While many adhesin bundles to the abdominal wall were observed. The stump appendicitis adhered to the ileum loop causing to small part of the ileum that was discolored and congested. Hence, appendectomy was done and the ileum loop was released, which led to resolved its congestion. Indeed, a definitive diagnosis was confirmed during exploratory laparoscopy, which revealed adhesion bands causing ileum obstruction (Figure-2).

The patient was hospitalized for one and three days in the intensive care unit and the surgery ward, respectively. Postoperative outcomes were favorable, with the resolution of symptoms and return to oral intake within a few days, and she was discharged in good general condition. The pathology report indicated the absence of appendicitis and any inflammation of resected tissue; therefore, ileum obstruction due to adhesive bundles followed by previous surgery (bariatric surgery) was considered as the final diagnosis. She was closely monitored during the three months to screen for recurrent adhesive band formation.

## Discussion

Ileum obstruction due to adhesion bands is a rare but noteworthy complication of sleeve gastrectomy [6]. Although effective treatment options exist, prevention is vital due to the potential morbidity associated with bowel obstruction and the need for surgical intervention [7]. Evidence suggests that surgeons performing sleeve gastrectomy should aim for meticulous surgical technique, minimizing tissue trauma, and implementing preventive measures to reduce the risk of adhesion formation [8][9].

Indeed, important strategies include meticulous surgical technique, using anti-adhesion barriers, minimizing tissue devascularization, and ensuring optimal healing and restoration of mesenteric perfusion [10]. Moreover, employing laparoscopic approaches with minimal tissue handling can also contribute to reducing the likelihood of adhesion bands [8].

Our patient mentioned a history of cesarean section and cholecystectomy approximately 15 and 20 years ago, respectively. Hence, it seems that in addition to the recent intervention (sleeve surgery), there are some risk factors for the formation of many adhesion bundles in our patient. However, the progressive adhesion of the appendix properly after sleeve surgery could exacerbate this condition and result in bowel obstruction.

In other words, the absence of acute appendicitis and consequently present ileal obstruction, could enhance the possibility of adhesions following recent surgery.

As mentioned before, there are no similar reports in the literature. Indeed, in the previous reports, ileum obstruction was followed by appendicitis or bowel obstruction due to open abdominal surgery. For instance, Capella et al., [5] reported 68 patients of bowel obstruction after laparoscopic gastric bypass surgery. They indicated that lack of adhesions and the resulting free displacement of the small bowel after laparoscopy appear to be the cause of this complication [5].

Currently, the use of barbed sutures has been widely adopted for use in gastric bypass surgeries to avoid the need for intracorporeal knot tying while maintaining tension and improving surgical efficiency [11]. Whilst barbed suture has been reported as safe with similar outcomes to traditional suture use in bariatric surgery there is a risk that the barbs on the tail of the suture can grasp other tissues and form band adhesions resulting in small bowel obstruction [11]. Hence, the exact mechanism for the formation of adhesive bundles of the appendix after gastric surgery that can lead to intestinal obstruction in the future has not been identified.

## Conclusion

The ileum obstruction resulting from adhesion bands following previous sleeve surgery is a rare complication. A high index of suspicion should be maintained for patients presenting with abdominal pain and a history of bariatric surgery. Prompt diagnosis via comprehensive imaging studies and definitive management with surgical intervention, such as laparoscopy, are important considerations. Additionally, surgeons should adopt preventive strategies during the primary procedure to minimize the likelihood of adhesion formation. Further research is warranted to explore additional measures that can improve patient outcomes while reducing the risk of adhesion-related complications.

## Conflict of Interest

There is no conflict of interest to disclose.
